# Historical first descriptions of Cajal–Retzius cells: from pioneer studies to current knowledge

**DOI:** 10.3389/fnana.2014.00032

**Published:** 2014-05-27

**Authors:** Vanessa Gil, Sara Nocentini, José A. del Río

**Affiliations:** ^1^Molecular and Cellular Neurobiotechnology, Institute for Bioengineering of Catalonia, Parc Científic de BarcelonaBarcelona, Spain; ^2^Department of Cell Biology, Faculty of Biology, University of Barcelona Barcelona, Spain; ^3^Centro de Investigación Biomédica en Red de Enfermedades NeurodegenerativasBarcelona, Spain

**Keywords:** neocortical development, pioneer neurons, radial glia, cortical hem, reelin, calretinin

## Abstract

Santiago Ramón y Cajal developed a great body of scientific research during the last decade of 19th century, mainly between 1888 and 1892, when he published more than 30 manuscripts. The neuronal theory, the structure of dendrites and spines, and fine microscopic descriptions of numerous neural circuits are among these studies. In addition, numerous cell types (neuronal and glial) were described by Ramón y Cajal during this time using this “reazione nera” or Golgi method. Among these neurons were the *special* cells of the molecular layer of the neocortex. These cells were also termed *Cajal* cells or *Retzius* cells by other colleagues. Today these cells are known as Cajal–Retzius cells. From the earliest description, several biological aspects of these fascinating cells have been analyzed (e.g., cell morphology, physiological properties, origin and cellular fate, putative function during cortical development, etc). In this review we will summarize in a temporal basis the emerging knowledge concerning this cell population with specific attention the pioneer studies of Santiago Ramón y Cajal.

## INTRODUCTION

Today it is generally accepted that Santiago Ramón y Cajal’s (1852–1934) studies, in particular the neuronal theory, should be considered the beginning of modern neurobiology (DeFelipe, 2002; Lopez-Munoz et al., 2006; De Carlos and Borrell, 2007). Thus, numerous aspects of Cajal’s activities, from a point of view of both scientific and academic, have been largely described in several manuscripts, reviews and books. Some of them focused on determining the relevance of Cajal’s technological advance to current neurobiology (DeFelipe and Jones, 1992; Jones, 2007). Today, when we characterize “translational research” as a robust pillar of an appropriate scientific strategy we should note, if we look at Cajal’s notes, that this vision is not new. In fact, it was also developed by Cajal, among others, during his scientific career. In science, the development of new methods and their implementation as translational tools for research is one of the mandatory in the face of new challenging issues regarding how to obtain relevant scientific data. As indicated, this vision was one of the greatest and most relevant contributions of Cajal to the “scientific method,” expanding the descriptive aspects of the method to a more deductive approach, as clearly demonstrated by his drawings. Indeed, this may be seen in the first stage of his research, between 1877 and 1887, and previous to the discovery of the “reazione nera” or Golgi method (Camillo Golgi, 1843–1926), in a visit to the private laboratory of Luis Simarro (1851–1921). Cajal equipped the Anatomy Department (Medical School) of the University of Valencia (1883) and Barcelona (1887) with optical microscopes. These pioneer microscopy units were the result of the privileged microscopic observations of the histological preparations of Aureliano Maestre de San Juan (1828–1890). In fact, we cannot describe the advances of Cajal without making a mention to the microscopic drawings and microphotographs, most of them developed at high magnification and using various histological methods, which meant a challenging issue at that time. However, we should not underestimate his deductive potential since in the hands of Cajal, the Golgi method showed a different neuronal organization from that described by Golgi and other scientists using the same method (Golgi, 1873). Another relevant aspect of Cajal’s studies was the description of the neuronal architecture by analyzing the development and then degeneration of the nervous system. Thus, during the period from 1887 to 1903, Cajal carried out intense and productive scientific activity, with the help of the Golgi method, in many descriptive aspects not only of mature nervous tissue but also of its development. In this review we would like to present some of the data that Cajal and colleagues published concerning a specific cell type located in the superficial layer of the developing cerebral cortex: the Cajal–Retzius cell. In addition we would like also to consider these results in light of current knowledge of this cell population.

## FIRST DESCRIPTIONS OF CAJAL–RETZIUS CELLS: FROM THE *CAJAL* CELLS OF RETZIUS TO THE HUMAN *RETZIUS* CELLS OF KÖLLIKER THROUGH THE *SPECIAL* CELLS OF CAJAL

**FIGURE 1:**
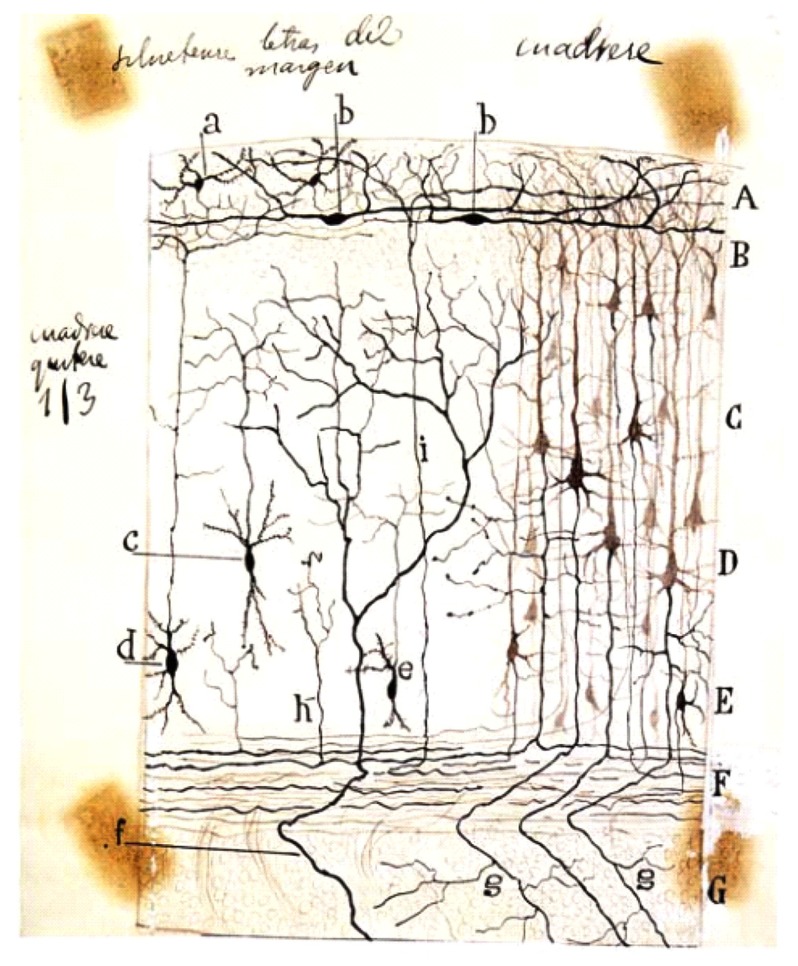
**Schematic drawing by Cajal of a Golgi-impregnated preparation of the cerebral cortex**. In this illustration, Cajal compiled some of his findings from small mammals (rabbit, mouse, etc.) reported between 1890 and 1891. Note both the presence of the polyhedral (or stellate) cells *(a)* and the horizontal fusiform cells *(b)* in the plexiform layer. **A**, plexiform layer; **B**, small pyramidal cell layer; **C**, medium pyramidal cell layer; **D**, giant pyramidal cell layer; **E**, polymorphic cell layer; **F**, white matter; **G**, striatum. Reproduced, with permission of the Inheritors of Santiago Ramón y Cajal, from Reference (Ramón y Cajal, 1923).

Cajal–Retzius cells have been extensively analyzed since Cajal first described them in 1890 (Ramón y Cajal, 1890). At that time, he was intrigued by the existence of a dense axonal plexus of nerve fibers that run horizontally to the surface of the cerebral cortex in the molecular layer. Some contemporary neuroanatomists described that these fibers were myelinated and suggested a putative origin for them. For example, Carlo Martinotti (1859–1918) suggested that they originated from the branches of pyramidal axons of the second and third cortical layer (Martinotti, 1890). However, the exact origin of them was unknown due mainly to the limitations of the histological techniques. Moreover, other scientists working on the structure of the neocortex described the presence of cells in layer I as well as the lamination of the human cortex using methylene blue staining without specific descriptions of these cells (Meynert, 1867). Taking advantage of the Golgi method, Cajal studied the composition of the marginal layer in newborn small mammals such as rabbit, cat, dog and rat (Ramón y Cajal, 1890). He observed that these fibers, in contrast to what was contained in Martinotti’s theory, arose mostly from two different cell types present in the same molecular layer: *polyhedral* and *fusiform* cells. The first were of medium size with four or five rough dendrite branches that extended in all directions, the axons of which ramified profusely in the most superficial part of the molecular layer. The second neuronal type was thinner and very elongated, with a smooth contour and with an ovoid soma and two opposed branches that extended horizontally over a considerable distance and finally bent and ascended to the cerebral surface. In their horizontal trajectory, their processes produced collateral processes or appendages which terminated in the upper portion of the molecular layer (**Figure 1**). But surprisingly, under the analysis of Cajal, these cells frequently showed two or three axons that came off the dendritic branches at a great distance from the cell body and then ran opposed and horizontally until they ramified in ascendant collaterals which afterwards turned so as to run horizontally, populating the entire marginal layer. This characteristic led Cajal to refer to them as *special cells*. Apart from this histological description (Ramón y Cajal, 1890), he took the risk of attributing to them a functional role and considered they might serve as a connection between pyramidal cells from distinct areas of the cortex. Thus, the arborizations of their nerve fibers contacted the apical dendrites of pyramidal cells; for this reason he also conferred upon them the name of *superficial cells of association* (Ramón y Cajal, 1890, 1891b).

Gustaf Retzius (1842–1919) identified these cells in embryos of diverse species (rabbit, cat, and dog) and called them *Cajal* cells (Cajal’sche Zellen; Retzius, 1893). The first description of these Cajal cells by Retzius was in parallel with the study of another cell type identified by Cajal as “interstitial” cells of the cortical white matter of dogs (Ramón y Cajal, 1891a, 1893). Indeed, Retzius described, in plate I of this publication of 1893, the presence of horizontally fusiform cells similar to those reported by Cajal. However, he failed to identify the same cell type in human fetuses. This led to Rudolph Albert von Kölliker’s (1817–1905) reserving the name of *Cajal* cells for mammals and employing the term *Retzius* cells for their human fetal homologues (Kölliker, 1896).

Some years later, the axon-like appearance of the majority of the cellular processes in these cells led Cajal to modify his previous opinion and to consider that these cells lacked a differentiation of processes into axons and dendrites and that they therefore shared the same morphological significance (Ramón y Cajal, 1897). However, the observations of Retzius in human fetuses and of Emilio Veratti (1872–1967) in rabbits (Veratti, 1897), in addition to his own observations obtained with new techniques (methylene blue and reduced silver nitrate methods), led him to give up the notion that these *special cells* possessed multiple axons and that only one behaved like a legitimate nerve fiber (Ramón y Cajal, 1904). Furthermore, due to the great morphological differences observed between cells from newborn children and fetuses, he concluded that in humans these *special cells* showed two stages: the fetal and the adult form. According to this theory, most of the fine ascending processes present in the fetal form were destined to atrophy in the days following birth, becoming almost completely absent in post-natal periods and therefore conferring upon these cells their characteristic adult form.

Cajal continued to study these *special* cells throughout his life in different areas of the human cortex such as visual, motor, olfactory, and acoustic areas, and also in different mammal species, birds, and reptiles, thereby performing the first comparative analysis of them (Ramón y Cajal, 1904, 1911; DeFelipe and Jones, 1988).

## A SECOND PHASE OF CAJAL–RETZIUS CELL ANALYSIS: CORTICAL LAMINATION STUDIES PREVAIL OVER CAJAL–RETZIUS CELL DESCRIPTIONS

Although Cajal and Retzius exhaustively characterized these cells, the great morphological complexity that they show in different species and in different developmental stages in addition to the random results obtained by the Golgi method have caused great confusion. Numerous studies have been directed to determining other aspects of the developing cortex instead of analyzing in greater detail the biology of Cajal–Retzius cells. For example, when analyzing descriptions of the white matter cells, intermediate zone/subventricular zone, and subplate, Cajal–Retzius cells appear in the published data as being cited but not studied in detail (Hatai, 1902; Von Economo and Koskinas, 1925; Aström, 1967). Even the appropriate name has become a matter of controversy, and they have received different names such as Cajal cells, Retzius cells and Retzius–Cajal cells (Fox and Inman, 1966; Konig, 1978). While the debate continued until recent years (Meyer et al., 1999; Fairen et al., 2002), the most widely accepted term today is Cajal–Retzius cells (henceforth CR cells).

## THE THIRD PHASE OF CAJAL–RETZIUS CELL STUDIES: GROWING INTEREST IN THE 1970s BECAUSE OF THE INFLUENCE OF THE BOULDER COMMITTEE

In 1970, a group of neurobiologists published a seminal review in Anatomical Record describing the basic principles of the development of the central nervous system (Boulder Committee, 1970). Although lacking some information (e.g., the subplate was not identified as a developmental layer in the manuscript and CR cells, although mentioned, were not included in the general scheme), the effort at summarizing most of the information obtained during several studies (mainly in humans) was very positive. In fact, the committee assigned the molecular layer a relevant role during cortical development. Following the publication of the manuscript, several studies analyzed the birthdates as well as the ultrastructure of the neuronal and glial populations described in the manuscript (Konig et al., 1977; Rickmann et al., 1977; Shoukimas and Hinds, 1978; Konig and Marty, 1981; Sievers and Raedler, 1981). But especially, special attention should be paid to the numerous studies developed by Miguel Marín-Padilla (1930) concerning the structure and development of the primitive plexiform layer/layer I in cats, hamsters, and humans (Marin-Padilla, 1971, 1988; Marin-Padilla and Marin-Padilla, 1982). In accordance with the original descriptions of Cajal and Retzius, Marin-Padilla, a follower in the trail laid down by Cajal, described the molecular layer as the first cortical lamina to develop during corticogenesis, characterized by the presence of a horizontal plexus of fibers with scattered primitive neurons. More relevantly, Marin-Padilla determined that both fibers and neurons are further split into the superficial (layer I) and deeper layers (layer VII, in human) by the appearance of the cortical plate (Marin-Padilla, 1978). Marin-Padilla’s descriptions of CR cells are based on the use of the Golgi method and are very similar to those reported by Cajal. He described CR cells in the fetal stage as cells with triangular, inverted pyramidal or fusiform cell bodies with two horizontal dendrites with ascending fine branches. The axon of the CR cells bifurcates in layer I and form the tangential fibers of Retzius (Marin-Padilla, 1988).

**FIGURE 2:**
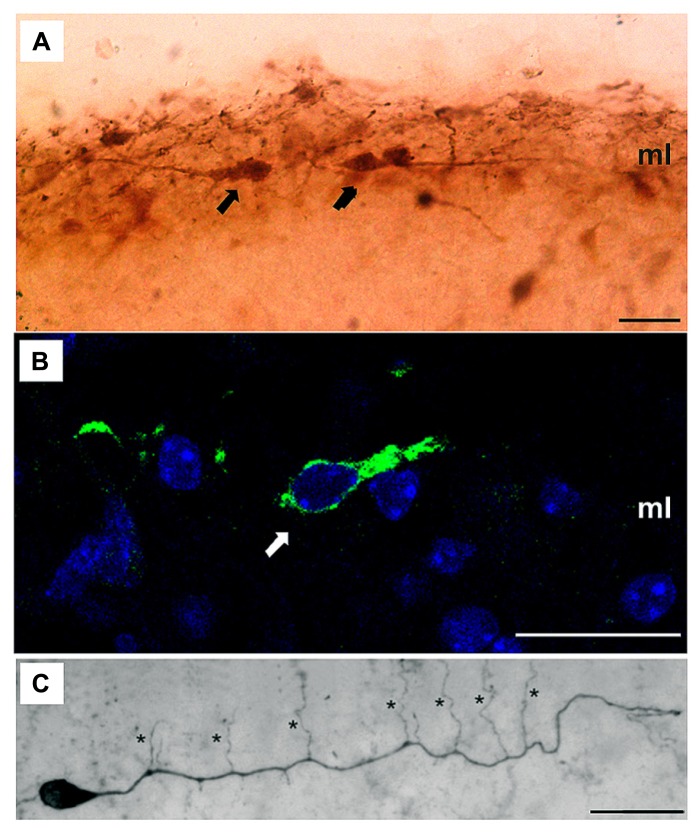
**Dendritic morphology of CR cells. (A,B)** High magnification microphotographs illustrating several CR cells (arrows) immuno-labeled with different specific markers such as Calretinin **(A)** or Reelin **(B)** in the molecular layer (ml); **(C)** Light microscopic image of a biocytin-filled CR cell from a P11 old CxBR4–eGFP mouse showing a single thick stem dendrite with vertically oriented side branches (marked by asterisks). Scale bars in **(A–C)** are 20 μm. Reproduced, with permission, from Anstötz et al. (2013).

The controversy about the different morphological features of these cells seems to be resolving gradually due to recent studies employing advanced techniques as *in vivo* two-photon imaging (Chowdhury et al., 2010) and the use of several markers such as Acetyl cholinesterase, Calretinin, Reelin, p73, CxCR4, etc. (Cabrera-Socorro et al., 2007; Anstötz et al., 2013; Ma et al., 2013; **Figure 2**). Nevertheless, the fact that these markers are not really specific for CR implies a huge disadvantage for studying these cells in detail, and therefore the characterization of a real specific marker should be one of the main goals of the researchers in this area.

## ORIGIN AND MIGRATORY ROUTES OF CR CELLS: A “ROAD RUNNER” IN THE DEVELOPING CORTEX

From the earliest studies, CR cells were thought to be generated in the ventricular region of the pallium (Meyer et al., 1998), while in the classical studies cited above they were described in different cortical regions. Classical descriptions do not discriminate between CR cell morphology and characteristics with respect to their origin. However, by using using Golgi impregnation (Marin-Padilla and Marin-Padilla, 1982) or other techniques such as tritiated thymidine (Parnavelas and Edmunds, 1983), it has been shown how CR cells undergo morphological changes to be transformed into resident layer I cells during cortical development in cats (Luskin and Shatz, 1985) and primates, including humans (Marin-Padilla, 1988). Thus, the incorporation of CR cells in the preplate came to be considered the result of radial migration during corticogenesis (Boulder Committee, 1970) and this was confirmed by recent studies (Gorski et al., 2002). But, recent evidences indicate that rodent CR cells are generated in different neuroproliferative regions of the telencephalon (see below) and that from these sources they migrate tangentially to completely populate the pallium. In summary, we can note that a small percentage of CR cells originate in the neuroepithelium while the majority of them originate outside the pallium. In 1998, an study focused in the subpial granular layer (SGL) in human fetus revealed that one population of Reelin-negative granule neurons from, after arriving in the marginal zone (MZ), differentiates into Reelin-producing neurons with CR morphology (Meyer and Goffinet, 1998). Some years later, analyzing the development and organization of layer I in macaque monkey, it was confirmed that SGL might be a site of origin for late-generated CR cells. Moreover, it was hypothesized that the olfactory primordium gave rise to the SGL in monkey, thereby putting this zone forward as another source of CR cells (Zecevic and Rakic, 2001). An additional source of CR cells was identified on the basis of p73 expression in 2002. In human fetus, at eight gestational weeks, a mediolateral gradient in the density of p73/Reelin-positive neurons in the neocortical MZ suggests that a subset of CR cells migrates tangentially from the cortical hem and taenia tecta (Meyer et al., 2002). The cortical hem as a source for CR in mouse was demonstrated using the IG17 transgenic mouse and *in utero* electroporation (Takiguchi-Hayashi et al., 2004). Recent studies have reported that cortical hem-derived CR cells mainly populate the dorso-medial regions of the cortex (Gu et al., 2011). Furthermore, in mice in which the cortical hem has been ablated, the MZ contains very few or no cells (neither CR nor other cell types) as well as very low levels of Reelin (Yoshida et al., 2006). In 2005, it was proved the existence of two previously unknown sites of origin for two distinct subsets of CR cells: the ventral pallium at the pallial–subpallial boundary (PSB) and the septum (Bielle et al., 2005). Using a knock-in strategy, combined with DiI labeling, they followed the fate of the progeny of *Dbx1*-derived cells through their entire lifespan. These cells gave rise to Reelin-positive neurons and became CR cells in the post-natal cortex with different characteristics (Bielle et al., 2005). The generation of *Dbx1*-derived cells seems to occur a bit earlier in the septum than in PSB. Moreover, cells migrated from the septum to the medial and piriform cortex and did not express Calretinin, whereas from the PSB they migrated to the dorso-lateral and piriform cortex and expressed Calretinin. More recently, the thalamic eminence (TE) (Takiguchi-Hayashi et al., 2004; Cabrera-Socorro et al., 2007; Meyer, 2010) and the “amygdalar hem” have been proposed as additional putative sites of origin (Meyer, 2010). In fact, in rodent TE-generated cells express markers of CR cells such as Calretinin (Abbott and Jacobowitz, 1999), DeltaNp73 (Tissir et al., 2009), and low levels of Reelin (Meyer, 2010). Although their final destination is unknown, they may reach the di-telencephalic sulcus and amygdala. Immediately caudal to the TE, there is another putative source of CR, the “amygdalar hem,” a small triangular area of neuroepithelium that connects the corticomedial amygdala to the choroid plexus at E12 in mice and which is also known as the “strionuclear neuroepithelium” (Altman and Bayer, 1995); cells originating in this area express high levels of Reelin when they are near the pial surface and therefore might represent CR cells whose final destinations are the amygdala and entorhinal cortex (Meyer, 2010). In summary, the cortical hem is the main source for CR cells but they are also produced in several sites such as SGL, taenia tecta, PSB, septum, TE, and the amygdalar hem.

Nowadays, we may affirm that CR cells originate in various focal sources in the developing brain, although we may not rule out the possibility that additional origin sites might exist. This multi-zonal production of CR may guarantee complete coverage of the cerebral cortex. Moreover, the various subtypes of CR cells generated at different sites intermingle in the cortex, in a way that cortical areas present a different proportion of distinct CR subtypes. This might contribute to determining area-specific properties (Bielle et al., 2005). At this point, two recent specific discoveries should also be noted: first, the distribution of CR cells in layer I depends on inhibitory cell-cell mechanisms (Villar-Cervino et al., 2013), and second, the distribution of CR cells is largely associated with their interaction with radial glia (Kwon et al., 2011). Finally, we would also like to remark that, probably, all the controversy generated around CR cells since they were first described by Cajal is due to the fact that they come from different sites what produces different CR subpopulations with specific morphological and physiological characteristics. Therefore, in order to achieve a thorough understanding of CR cells, researchers need to develop strategies that combine birth-dating and tracing studies with specific markers.

## ROLE OF CAJAL–RETZIUS CELLS IN RADIAL NEURONAL MIGRATION

The mammalian neocortex is a highly ordered structure in which different types of neurons are arranged by tangential and radial migration during embryonic development to form the final laminated organization. This elaborate assembly is accomplished in distinct steps. The first step is the formation of the preplate, composed of a superficial plexus of corticopetal nerve fibers and a heterogeneous population of post-migratory cells, including CR cells, interneurons, and future subplate neurons (Bielas et al., 2004; Bystron et al., 2008). In the second step, waves of post-mitotic neurons exit the ventricular zone and move in a radial direction toward the pial surface, where upon they split the preplate into the MZ (above), which would contain the CR cells, and the subplate (below), thus establishing another cellular band known as the cortical plate. This new layer of cells contributes to layers II-VI of the cortex in rodents (Angevine and Sidman, 1961; Berry and Rogers, 1965; Rakic, 1972). In 1995, it was described how CR cells are responsible for the correct lamination of the neocortex through the secretion of an extracellular protein called Reelin (D’Arcangelo et al., 1995; Ogawa et al., 1995). In mice lacking Reelin the preplate does not split properly into the MZ and subplate. Consequently, this structure constitutes a “superplate” in the most superficial region of the cortex. Cortical plate neurons accumulate beneath the superplate; young neurons cannot migrate outward by passing across pre-existing cell layers. This results in an inverted pattern of neuronal positioning in all laminated structures (neocortex, cerebellum and hippocampus) as well as in subcortical structures such as the olfactory bulb, inferior olivary complex, and facial nucleus (Caviness and Sidman, 1973; Caviness, 1982). The list of molecular partners of the Reelin pathway is continuously increasing and it has been shown that the integrity of the Reelin-signaling cascade is essential for the correct positioning of cortical plate neurons and disruption of any of its components leads to failure of radial migration (Gao and Godbout, 2013).

Recently, the comparison of Reelin patterns between amniote species showing some degree of cortical lamination (mammals and lizards) and those with no obvious pallial cytoarchitectonic condensation at all (turtles and birds) led to a “Reelin hypothesis” for cortical developmental evolution, with the condensation of Reelin-expressing cells being a key feature of the establishment of a sophisticated laminated pattern. In fact, the relevance of developing layer I during cortical evolution was hypotethized some years earlier (Marin-Padilla, 1978). These comparative data point up the importance of the Reelin pathway, and hence of CR cells, in the morphogenesis and cytoarchitecture of pallial structures (Abellan et al., 2010). However, we should take into account that Reelin is not uniquely expressed by CR cells but also by interneurons (Alcantara et al., 1998), and therefore we should not confer the main role in orquestrating the radial neuronal migration to CR cells, even though Reelin-positive interneurons have only been detected at post-natal stages (Alcantara et al., 1998; Ma et al., 2013).

Moreover, different strategies addressed to eliminating the presence of CR cells have questioned its importance in cell migration. These methods highlight the relevance of CR cells in radial glia maintenance and function (Soriano and del Rio, 2005). For example, local application of a toxic agent to newborn mouse cortex ablates CR cells and disrupts cell migration to layers II/III, causing radial glia to change to astroglia (Super et al., 2000). And in mutants for p73 and Emx1/Emx2 in which there is an absence of CR cells, the cortical pattern is altered although preplate partition and cortical plate formation are not disturbed (Shinozaki et al., 2002; Meyer et al., 2004). In contrast, the ablation of the cortical hem, the predominant source of CR cells, did not produce the inverted lamination observed in the *reeler* mutant (Yoshida et al., 2006). In line with this unexpected result, some studies have suggested that, rather than CR cells, it is the integrity of the pial basement membrane and meningeal cells that is crucial for correct cortical histogenesis (Halfter et al., 2002; Beggs et al., 2003). Nevertheless, another link has been recently reported between CR cells and radial migration through the immunoglobulin-like adhesion molecule Nectin1. In this study, authors described that these cells express Nectin1 which interacts with Nectin3, present in projection neurons, and that this interaction is critical for radial migration (Gil-Sanz et al., 2013). Interestingly, these molecules belong to the Reelin signaling pathway which indicates, once again, a role of CR cells and Reelin in this process.

## POST-NATAL FATE OF CR CELLS: RODENTS VS. PRIMATES

Some studies have suggested that CR cells undergo a morphological change in order to become resident interneurons of layer I in adult neocortex (Parnavelas and Edmunds, 1983). Others have proposed that the decrease in CR neuron density is caused by dilution from the expansion of the cortex during development, without a clear morphological transformation (Marin-Padilla, 1990). However, the most widely accepted theory is that most of the cells are destined to disappear by adulthood and undergo cell death (Derer and Derer, 1990). In rodents, signs of CR neuron degeneration begin in the second post-natal week, as evidenced by retraction of thin appendages from their main dendrite, swelling of the endoplasmic reticulum, and darkening of the cytoplasm (Derer and Derer, 1990; Soriano et al., 1994; del Rio et al., 1995). This CR cell loss has also been reported to occur directly after migration in monkeys and humans (Zecevic and Rakic, 2001; Abraham and Meyer, 2003). In these previous studies it was already reported that in rodents nearly 95% of CR cells present at birth may disappear before P10–P14 (del Rio et al., 1995; Soda et al., 2003) and these data have recently been recalculated analyzing identified CR neurons that express the green fluorescent protein under the control of different promoters such as the early B-cell factor 2 (Ebf2) or CxBR4 promoters (Chowdhury et al., 2010; Anstötz et al. 2013). Researchers concluded that most CR cells die progressively by apoptosis from P7 onwards and only a small fraction (3–4%) present at birth survive into adulthood (Chowdhury et al., 2010; Anstötz et al., 2013; Ma et al., 2013). However, in contrast to the nearly complete elimination of CR cells in the post-natal neocortex, 25% of CR cells appear to survive in the hippocampus of adult animals (Super et al., 1998; Anstötz et al., 2013), reflecting a differential role between neocortical and hippocampal CR cells in the adulthood. Therefore, neocortical CR cells appear at early stages of embryonic life, they increase their density at the first post-natal week and decrease from there until reaching minimum levels.

## CR CELLS DURING POST-NATAL LIFE

**FIGURE 3:**
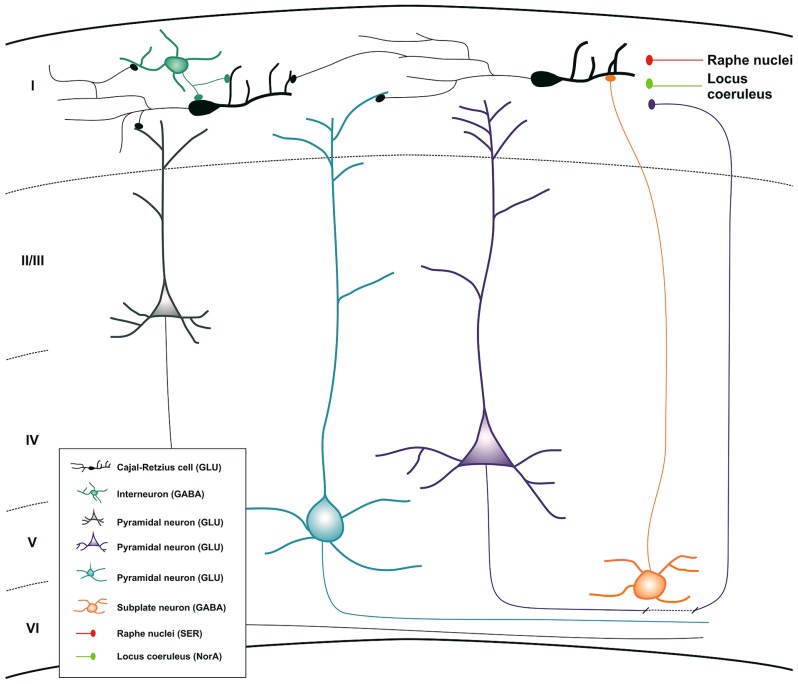
**Schematic drawing summarizing all the inputs and outputs connections of CR cells during neocortical development**. Inset map shows the distinct cell populations involved in the process with their principal neurotransmitters. Abbreviations: GLU, glutamate; GABA, gamma-aminobutyric acid; NorA, noradrenalin; SER, serotonin.

There is no plausible explanation for the presence of CR cells during embryonic life and their atrophy and eventual disappearance shortly after birth. Historically, as we have indicated above, researchers have focused their efforts in studying the role of CR cells in cortical migration at the prenatal period. However, it would be interesting to think about a putative function of CR cells during early post-natal stages, when CR cells reach their highest density (between P3 and P7) (Anstötz et al., 2013) and the cortical lamination has been already completed. The first post-natal week is a critical period for the maturation of interneurons and pyramidal cells in the neocortex and for the establishment of their final connections. Several studies have described that CR cells show spontaneous activity and that they could belong to an early cortical network that controls the maturation of the cerebral cortex (Marin-Padilla, 1998; Aguilo et al., 1999; Radnikow et al., 2002). On the other hand, it was already demonstrated that this cell population was one the earliest functional neurons in the developing human brain when a study in 1968 described the presence of acetylcholinesterase activity in their cytoplasm in 4-month-old human fetuses (Duckett and Pearse, 1968). Moreover, they are related to interneurons and neurons via input and output connections, receiving GABAergic, serotonergic, glutamatergic, and noradrenergic inputs and sending glutamatergic information (**Figure 3**; del Rio et al., 1995; Radnikow et al., 2002; Janusonis et al., 2004; Chowdhury et al., 2010; Myakhar et al., 2011; Anstötz et al., 2013). The morphological (long-range horizontal axonal projection) and electrical features of the CR cells and their synaptic input–output relationship at this period would facilitate the stabilization of interneurons and pyramidal dendritic trees suggesting that CR cells can integrate information in layer I and send projections to target neurons to facilitate the formation of the neocortical network during very early stages of development, lending support to Cajal’s associational theory (Molliver et al., 1973; Konig et al., 1975; Bradford et al., 1977; Konig and Marty, 1981; Rickmann and Wolff, 1981). After the early neocortical network is established, most of the CR cells would degenerate and die. Furthermore, no functional role of the small population that remains in the adulthood has been offered.

## CR CELLS THROUGHOUT EVOLUTION

As indicated above, Cajal and Retzius described, for the first time, CR cells in the MZ of the fetal and early post-natal neocortex in humans, small mammals (rabbit, cat, dog, rat, and mouse), birds, and reptiles. Since then, CR cells have been described in mammalian and non-mammalian vertebrates (Tissir et al., 2003; Cabrera-Socorro et al., 2007; Abellan et al., 2010). Although CR cells are present in all amniotes, the Reelin signal in CR cells increases in mammals and is even higher in primates including humans (Meyer and Goffinet, 1998). In mammals, it has been proposed that the increasing proportion of p73-positive CR cells may contribute to the evolutionary amplification of Reelin-signal in the MZ during development (Cabrera-Socorro et al., 2007). Moreover, p73 expression seems to be related to the prolonged survival and increased differentiation of dendritic and axonal processes of CR. Observing CR cells in lizard, mouse and human, it was proposed that p73 may play a role in the acquisition of complexity in CR cells during evolution. In fact, CR cells are rudimentary in lizards, relatively simple in mice, and more complex in primates (Cabrera-Socorro et al., 2007).

The abundance of CR cells seems to correlate with the size of the cortical hem, which has been demonstrated to be small in sauropsids such as crocodiles (Tissir et al., 2003), lizards (Goffinet et al., 1999) and chicks (Cabrera-Socorro et al., 2007) and maximal in humans (Cabrera-Socorro et al., 2007; Villar-Cervino and Marin, 2012). The varying size of the cortical hem influencing the number of CR cells could be understood as relevant factors in the evolution of cortical regions across vertebrates. An additional difference between primates and other vertebrates is that in the former at least two types of Reelin-producing cells have been described: large CR cells and at later developmental stages smaller SGL cells (Zecevic and Rakic, 2001). This second cellular type is thought to compensate for the progressive loss of CR cells during the long period of corticogenesis in primates (Zecevic and Rakic, 2001). In another study, it was reported a differential expression of LIM-homeodomain (LIM-hd) factors in primates, birds, and rodents (Abellan et al., 2010). They proposed that the expression of a larger repertoire of LIM-hd transcription factors in CR cells may correlate with their diversification and morphological complexity. These factors are assumed to convey higher molecular diversity and the possibility of promoting the emergence of novelties. Indeed CR cells in primates express at least four LIM-hd factors while in rodents the figure is only two. In chicks, none of these factors were found with the exception of Lhx5 in a small zone of the cortical hem (Abellan et al., 2010). In summary, we can conclude that the different abundance and complexity of the different CR cells subpopulations, which are characterized by both their specific origin and molecular profile, is involved in the level of complexity of the neocortical structure.

## CONCLUDING REMARKS

Numerous efforts have been targeted to understand the biology of the CR cells population since it was first described by Cajal in 1890. Paradoxically, when we analyze carefully all the studies reported during more than a century, we realize that the most important features of these cells (morphological and physiological properties) were already indicated by Cajal by employing very rudimentary methodological techniques. This fact points out the importance of his work for the current Neurobiology knowledge. Nowadays, their morphology and electrical properties are better known and we can also specify that they come from several origin sites, although the cortical hem is the most important source. Apart from this, evidence shows that CR cells exert different functions throughout the distinct periods of development, thus regulating the radial neuronal migration during prenatal life and possibly facilitating the cortical network assembly in the post-natal stage. However, to fully understand the exact role of the CR cells in the building of the cerebral cortex, new strategies that may allow the characterization of the different CR cells subsets are needed.

## Conflict of Interest Statement

The authors declare that the research was conducted in the absence of any commercial or financial relationships that could be construed as a potential conflict of interest.
